# Parosteal Osteosarcoma: A Benign-Looking Tumour, Amenable to a Variety of Surgical Reconstruction

**DOI:** 10.1155/2020/4807612

**Published:** 2020-05-28

**Authors:** Yogi Prabowo, Achmad Fauzi Kamal, Evelina Kodrat, Marcel Prasetyo, Samuel Maruanaya, Toto Suryo Efar

**Affiliations:** ^1^Department of Orthopaedic & Traumatology, Faculty of Medicine Universitas Indonesia/Cipto Mangunkusumo General Hospital, Jl. Salemba Raya No. 6, Jakarta Pusat 10430, Indonesia; ^2^Department of Anatomical Pathology, Faculty of Medicine Universitas Indonesia/Cipto Mangunkusumo General Hospital, Jl. Salemba Raya No. 6, Jakarta Pusat 10430, Indonesia; ^3^Department of Radiology, Faculty of Medicine Universitas Indonesia/Cipto Mangunkusumo General Hospital, Jl. Salemba Raya No. 6, Jakarta Pusat 10430, Indonesia

## Abstract

Osteosarcoma arising from cortical surface is classified into parosteal, periosteal and high-grade surface osteosarcoma. Along the spectrum, parosteal osteosarcoma occupies the well-differentiated end. It is a relatively rare disease entity, comprised only 4% of all osteosarcomas and barely reported in the literature. The objective of this study is to describe cases of parosteal osteosarcoma as well as a variety of treatment options amenable to such entity. Six cases of parosteal osteosarcoma were identified based on histopathological reports in a tertiary referral hospital in Jakarta, Indonesia between January 2001 and December 2019. The mean age was 29.8 years old; four of them (66.7%) were male. Distal end of femur was the most commonly involved bone (five cases, 83.3%). The patients were treated with wide excision followed by several different reconstruction methods: replacement with endoprosthesis, extracorporeal irradiation, knee arthrodesis, or prophylactic fixation. One of our patients presented with dedifferentiated component, and therefore was treated by limb ablation. While two cases died of pulmonary metastasis, other patients reported fair to excellent functional outcome.

## 1. Introduction

Osteosarcoma is a primary bone malignancy characterized by malignant cells of mesenchymal origin depositing immature osteoid matrix. It is one of the most common neoplasms of the musculoskeletal system, accounting for approximately one-fifth of all primary malignant bone tumours. Osteosarcoma encompasses various types of bony lesions, with the typical manifestation which is a high-grade sarcoma arise intramedullary. A few of the disease entities arise on the outer cortical surface; such lesions are termed surface osteosarcomas which are further divided into parosteal osteosarcoma, periosteal osteosarcoma, and high-grade surface osteosarcoma [1, 2]. Parosteal osteosarcoma represents the well-differentiated end of surface osteosarcoma, which typically consists of a slow-growing lesion that assumes a significantly better prognosis than conventional osteosarcoma [3]. However, it rarely manifests as a dedifferentiated fashion, which is usually more abrupt and is associated with poorer prognosis.

Parosteal osteosarcoma has been rarely reported in the literature. To date, there are no data regarding this tumour in our region. The objective of this study is to describe cases of parosteal osteosarcoma as well as a variety of treatment options amenable to such entity.

## 2. Materials and Methods

We searched through the clinicopathological reports and histopathological archives of patients diagnosed with parosteal osteosarcoma who visited Cipto Mangunkusumo Hospital, Jakarta, Indonesia, between the period of January 1999 and August 2018. Data regarding age, sex, tumour site, duration of symptoms, neoadjuvant chemotherapy, surgical treatment, and presence of local recurrence or metastasis were recorded. The patients were further followed up and asked to visit our Musculoskeletal Oncology outpatient clinic where the musculoskeletal tumour society score (MSTS) was evaluated. All patients agreed to participate in the study and provided written consent prior to the publication.

## 3. Results

**Figure 1:**
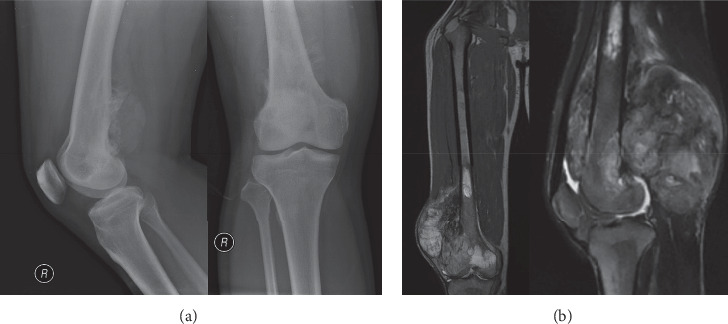
Radiographical appearance of parosteal osteosarcoma showing radiodense sessile lesion on the bone cortical surface, localized in the popliteal fossa (a). Magnetic resonance of T2-weighted coronal and sagittal images (b) depicting the heterogenic solid mass of distal femoral epimetaphysis with posterolateral soft tissue expansion. High signal intensity on the proximal marrow suggested intramedullary metastasis.

**Figure 2:**
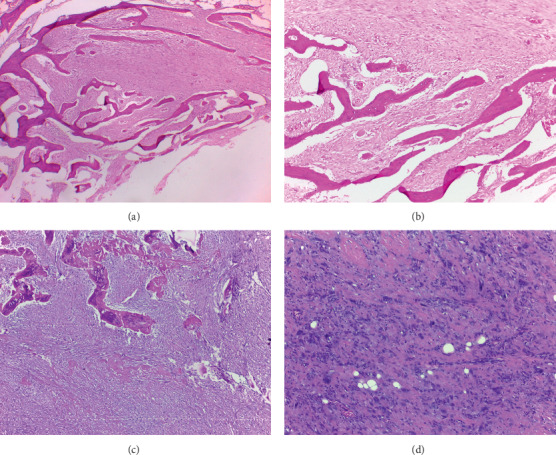
Histopathological appearance of parosteal osteosarcoma typically shows interconnected bony trabeculae with fibrous stromal tissue (H & E 40x A and 100x B. Rarely, parosteal osteosarcoma could also manifest as dedifferentiated type, which demonstrates irregular bony trabeculae (H & E, 40x C) and cellular foci of high-grade tumour (H & E, 100x D).

Six cases of parosteal osteosarcoma were identified (Table 1). We followed up the patient for a mean of 35.2 months. The mean age was 29.8 years old; four out of six patients were males. In five (83.3%) patient, the tumour arose on the distal end of the femur, while in one (16.7%) patient it was located on the distal end of humerus. In all cases, the diagnosis was established in the clinicopathological conference. The majority of the patients demonstrated typical radiographic appearance: a radiodense sessile lesion on the bone cortical surface arising from the metaphyseal region, localized eccentrically on the popliteal fossa. The larger the tumour, the more diffuse patterns it tended to encircle the host bone (Figure 1(a)). MRI was ordered for all patients to assess the degree of soft tissue extension, involvement of neurovascular structures, and presence of the intramedullary lesion (Figure 1(b)). We did not encounter any significant difficulty in diagnosing the tumour since all but one case showed typical histopathological appearance of parosteal osteosarcoma, which was either parallel pattern or interconnected bony trabeculae with fibrous stroma (Figures 2(a) and 2(b)).

We put an emphasis for case no. 6, who was a 37-year-old female who was diagnosed as osteochondroma elsewhere and twice underwent simple excision of the thought-to-be-benign tumour. Upon admission to our center, the tumour grew at an alarming speed. Without any histopathological report or slide from previous hospital, the tumour was biopsied and demonstrated appearance of irregular bony trabeculae with cellular foci of high-grade tumour (Figures 2(c) and 2(d)), indicating a dedifferentiated pattern of parosteal osteosarcoma.

While all patients received a wide margin of tumour resection, this series showed that parosteal osteosarcoma was amenable to a variety of reconstruction methods. Case no. 1 was treated with extracorporeal irradiation for the tumour on the distal humerus. Unfortunately, at 30-month follow-up, the patient died from lung metastasis that was unrecognized at the time of diagnosis.

**Figure 3:**
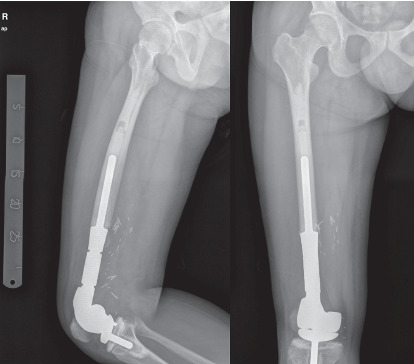
Postoperative radiographs of a 29-year-old male patient (case number 3) who underwent extra-articular resection and replacement with endoprosthesis.

Moreover, our case nos. 2 and 3 underwent joint replacement with endoprosthesis (Figure 3). However, patientno. 2 had a troublesome complication of periprosthetic joint infection. After device removal along and several debridement, the subject currently had the prosthesis reimplanted and had been infection-free for more than a year. While patient no. 3 achieved an excellent functional outcome, his counterpart recorded a fair result.

**Figure 4:**
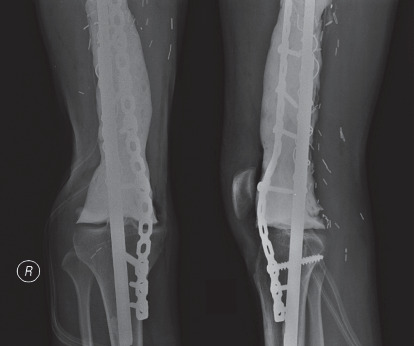
Postoperative radiographs of a 23-year-old male patient (case number 4) who underwent intra-articular resection and knee arthrodesis using the metallic-plus-bone-cement method.

Out of the six patients, only case no. 4 was administered a regimen of neoadjuvant chemotherapy because of the patient manifested lung and intramedullary metastasis at the time of diagnosis. After tumour excision, he underwent knee arthrodesis using Küntscher intramedullary nail, plate, and bone cement (Figure 4). He died from the disease at 18 months of follow-up.

**Figure 5:**
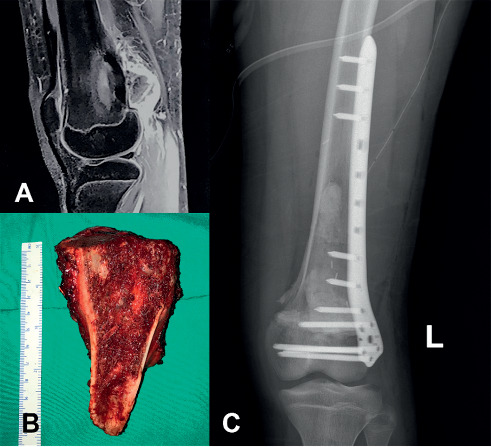
A sagittal T1-weighted fat-saturated MR image of a 14-year-old boy (case number 5) (a) demonstrating a broad-based tumour on the metaphyseal region of the distal femur. The patient was treated with hemicortical resection and prophylactic fixation using the plate and screw. Intraoperative gross pathology of the tumour (b) and postoperative radiograph (c) are presented.

Furthermore, case no. 5 was a 14-year-old boy who was brought to medical attention early when the tumour was relatively small in size. The patient underwent hemicortical excision and prophylactic fixation using a plate (Figure 5). Currently, that patient was free from the disease with a satisfying level of functional outcome.

**Figure 6:**
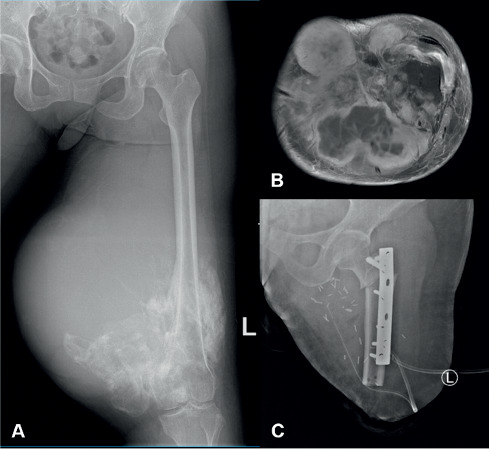
A 37-year-old female (case number 6) manifested local recurrence after misdiagnosed as osteochondroma and underwent multiple simple excisions. Preoperative radiograph of the left femur depicting an expansive juxtacortical bone tumour with malignant characteristics (a). An axial T2-weighted MR image demonstrating tumour extension to posterior and medial compartment of the femur, with displacement and invasion of major neurovascular bundles (b). Showing a dedifferentiated pattern on core biopsy, the patient finally underwent transfemoral amputation and tibial augmentation (c) at our center.

Lastly, our case no. 6, who demonstrated a dedifferentiated pattern of parosteal osteosarcoma, was performed high transfemoral amputation and augmentation using the patient's ipsilateral ablated tibia (Figure 6). At the last follow-up, the patient recorded a fair MSTS while still free from the disease. Having a more malignant pattern of the tumour, the patient did not develop lung or intramedullary metastasis.

## 4. Discussion

Parosteal osteosarcoma is a relatively rare bone tumour. It is reported that, in our national referral hospital, the incidence of osteosarcoma was 16.8 cases per year [4]. From this series, we identified six patients with parosteal osteosarcoma over a period of twenty years. Based on data from our center, parosteal osteosarcoma accounts for 1.79% of all osteosarcomas, which was slightly lower in comparison with previous findings [5].

Characteristics of patients in the current study such as age, sex, and tumour location were also in accordance with the big picture from literature. Parosteal osteosarcoma has slight female predominance and more frequently encountered in the third decade of life [1]. Posterior aspect of distal femoral metaphysis is the most common site followed by proximal tibial and proximal humeral metaphysis [6]. Those three locations combined account for more than 80% of all cases of parosteal osteosarcoma [5].

Parosteal osteosarcoma is a slow-growing tumour arising from the outer layer of the periosteum. It represents well-differentiated end of the spectrum of surface osteosarcomas. The tumour is relatively distinguishable from other types of osteosarcoma by a combination of radiographic and histopathological characteristics. Radiographically, it appears as a sessile, ossified, lobulated mass which attaches to the underlying bone via a broad pedicle but does not penetrate the cortex to involve the medulla. In addition, there was little or no periosteal new bone formation. Histologically, parosteal osteosarcoma typically presents with irregular bony trabeculae and bland-appearing spindle cells within the fibrous stromal tissue. Markedly, atypical cells and atypical mitoses are not present [7].

Those radiographical and histological characteristics can therefore be mistaken for benign lesions such as osteochondroma. They have similar predilections in age and tumour sites. Moreover, both tumours could be a sessile mass covered by a thin cartilage cap [1, 2]. One of our patients was mistakenly diagnosed as osteochondroma and was performed simple excision at a remote, rural health center. In our center, the patient manifested a dedifferentiated pattern of parosteal osteosarcoma, and given the clinical presentation, the involved limb had to be ablated. We augmented the stump using the amputated tibia to preserve some of the limb length. However, no matter how hard we strived, the outcome would obviously be more satisfying if the limb was salvaged and reconstructed in timely manner. In addition to osteochondroma, the appearance of parosteal osteosarcoma in its earlier stage could also be mistaken for several benign musculoskeletal tumours such as fibrous dysplasia, desmoplastic fibroma, or fibromatosis. In some cases, fibrous dysplasia presents as a protuberans, or exophytic, lesion which is characterized by irregular pattern of trabecular bone resembling Chinese alphabets. On the contrary, desmoplastic fibroma is predominated with spindle cell stroma and collagen bundles [1]. Especially when the tumour infiltrates surrounding soft tissues, parosteal osteosarcoma could also mimic soft tissue tumours such as fibromatosis [5]. Considering those difficulties, even if the suspected tumour is more likely to be a benign one, we strongly recommended proper and complete radiographical and histopathological investigations before establishing the preoperative diagnosis in a clinicopathological conference.

The cases of dedifferentiation that were discovered at the time of repeated excision after local recurrence have been reported elsewhere [8, 9]. On the contrary, incomplete excision almost inevitably leads to local recurrence [5, 10, 11]. Accordingly, in addition to comprehensive diagnostic strategy, the development of the dedifferentiated pattern also emphasized the importance of achieving a wide surgical margin at initial excision.

After tumour resection, the remaining bone defect could be managed by a variety of reconstructive surgeries. Lesions at the distal portion of a long bone, especially if the adjacent joint is involved, are best treated with endoprosthesis. However, the availability of the implants is relatively precarious in developing countries. If endoprosthesis is unavailable, extracorporeal irradiation followed by reimplantation (case no. 1) or resection arthrodesis using metallic plus bone cement (case no. 4) could be the alternative methods of surgical reconstruction. Both methods have been found to result in a reasonable functional outcome even in later stages of the disease [12].

Patients with earlier stages of parosteal osteosarcoma are amenable to cortical resection with or without prophylactic fixation. Our patient no. 5 with relatively localized tumour, minimal cortical involvement, and little soft tissue involvement was treated by hemicortical excision and prophylactic plating. On the contrary, patients in a very late stage of local tumour with local recurrence after several excisions (such as our case no. 6) should be treated with limb ablation. When the tumour is large and circumferential with neurovascular involvement, contaminated from previous biopsy or procedure, or could not be excised with a clear wide margin, ablation should be considered [5].

Regarding systemic treatment, we administered neoadjuvant chemotherapy only for case no. 4 who had lung metastasis at the time of diagnosis. Current chemotherapeutic agents offer little or no benefit parosteal osteosarcoma, with degree of tumour necrosis and disease survival after chemotherapy being unclear [1, 8, 13, 14]. However, several authors recommended the administration of chemotherapy in cases of dedifferentiated pattern [8, 14, 15], medullary involvement [11, 16], or lung metastasis [14].

Parosteal osteosarcoma carries much better prognosis than the classical, high-grade osteosarcoma. Four of our patients lived with no evidence of disease for a median follow-up of 41 months. The five-year disease-free survival of parosteal osteosarcoma is approximately 90% [17–19]. In this study, two patients with lung metastasis died after 30 and 18 months. Lung metastasis was associated with poorer overall survival, while in turn, the longer the tumour duration and higher histological grade were associated with an increasing rate of intramedullary involvement and distant metastasis as well as a poorer clinical outcome. Furthermore, the dedifferentiated type of parosteal osteosarcoma also led to relatively poorer prognosis: several studies [5, 9] have demonstrated poor survival similar to that of conventional osteosarcoma, while other studies [8, 20] reported slightly better prognosis. Intramedullary extension, or sometimes called as intramedullary metastasis, is still unclear in terms of whether it affects the overall prognosis [5].

## 5. Conclusion

Presented as a benign-looking tumour, parosteal osteosarcoma could be mistaken for severalmusculoskeletal lesions such as osteochondroma. Consequently, the diagnosis of parostealosteosarcoma should be established carefully on the basis of clinical, radiological, andhistopathological findings. Our series further emphasized that inadequate diagnosis or unplanned excision could lead to local recurrence, which was associated with the development of the more aggressive dedifferentiated pattern. In addition to wide-margin excision, the tumour was amenable to a wide range of reconstruction modality with fair to excellent outcome.

## Figures and Tables

**Table 1:** Characteristics of the patients.

No	Age	Sex	Site	Duration of symptoms (months)	NAC	Surgical treatment	Recurrence or metastasis	Survival	Follow-up (months)	MSTS
1	51	M	DH	18	No	ECI	LM	DOD	30	—

2	25	F	DF	36	No	EP	PJI	NED	73	19

3	29	M	DF	42	No	EP	None	NED	56	30

4	23	M	DF	60	Yes	Arthrodesis	IME and LM	DOD	18	—

5	14	M	DF	5	No	Prophylactic fixation	None	NED	25	31

6	37	F	DF	12	No	TFA	None	NED	9	15

NAC, neoadjuvant chemotherapy. MSTS, musculoskeletal tumour society score. F, female. M, male. DH, distal humerus. DF, distal femur. ECI, extracorporeal irradiation. EP, endoprosthesis. TFA, transfemoral amputation. LM, lung metastasis. PJI, periprosthetic joint infection. IMM, intramedullary extension. DOD, died of disease. NED, no evidence of disease.

## Data Availability

The data used to support the findings of this study are already included within the article.
